# The first taxonomic and functional characterization of human CAVD-associated microbiota

**DOI:** 10.15698/mic2023.02.791

**Published:** 2023-01-13

**Authors:** Lavinia Curini, Brunilda Alushi, Mary Roxana Christopher, Simone Baldi, Leandro Di Gloria, Pierluigi Stefano, Anna Laganà, Luisa Iannone, Herko Grubitzsch, Ulf Landmesser, Matteo Ramazzotti, Elena Niccolai, Alexander Lauten, Amedeo Amedei

**Affiliations:** 1 Department of Clinical and Experimental Medicine, University of Florence, 50139 Florence, Italy.; 2 Department of Cardiology, Campus Benjamin Franklin, Charité Universitätsmedizin Berlin, and German Centre for Cardiovascular Research (DZHK); Department of Interventional Cardiology, Klinik Vincentinum Augsburg, Germany.; 3 Department of Cardiology, Campus Benjamin Franklin, Charité Universitätsmedizin Berlin, and German Centre for Cardiovascular Research (DZHK).; 4 Department of Biomedical, Experimental and Clinical Sciences “Mario Serio”, University of Florence, Florence, Italy.; 5 Cardiac Surgery, Careggi University Hospital, 50134 Florence, Italy.; 6 Berlin Institute of Health; Department of Cardiology, German Heart Centre Berlin (DHZB).; 7 Department of Cardiology, Campus Benjamin Franklin, Charité Universitätsmedizin Berlin and German Centre for Cardiovascular Research (DZHK); Berlin Institute of Health.; 8 SOD of Interdisciplinary Internal Medicine, Azienda Ospedaliera Universitaria Careggi (AOUC), 50139 Florence, Italy.

**Keywords:** aortic valve disease, valve calcification, microbiota, immune response, T cells

## Abstract

**Introduction:**

Calcific aortic valve disease (CAVD) is the most common heart valve disorder, defined by a remodeling multistep process: namely, valve fibrosis with its area narrowing, impaired blood flow, and final calcification phase. Nowadays, the only treatment is the surgical valve replacement. As for other cardiovascular diseases, growing evidence suggest an active role of the immune system in the calcification process that could be modulated by the microbiota. To address this point, we aimed to investigate and characterize, for the first time, the presence of a valve microbiota and associated immune response in human CAVD.

**Method:**

Calcified aortic valve (CAV) samples from twenty patients (11 from Germany and 9 from Italy) with diagnosis of severe symptomatic CAVD were used to assess the presence of infiltrating T cells, by cloning approach, and to characterize the valve microbiota, by 16S rRNA gene sequencing (NGS).

**Results:**

We documented the presence of infiltrating T lymphocytes, especially the T helper subset, in CAV samples. Moreover, we found a tissue-associated microbiota in freshly collected CAV samples, which was significantly different in Italian and German patients, suggesting potential correlation with other cardiovascular risk factors.

**Conclusion:**

The presence of microbiota in inflamed CAV samples represents the right trigger point to explain the valve calcification process, encouraging further studies to explore the potential link between bacteria and adaptive immune response and to define the critical role of local microbiota-immunity axis on CAVD development.

## INTRODUCTION

Calcific aortic valve disease (CAVD) is a chronic disorder that involves a mineralization of the aortic valve (AV) tissue. CAVD is considered the most frequent heart valve disorder represented by a progressive thickening of the AV leaflets that leads to increased shear and mechanical stress on the left ventricle, resulting in a severe aortic stenosis and by an ectopic mineralization of the AV tissue [1].

In the past, calcification process was considered a passive degenerative process but, recent and increasing data suggest an active progression triggered by the valve interstitial cells (VICs), the major cellular AV component [2]. In detail, the VICs mineralization is promoted by immune system responsivity against various stimuli of different nature [3]. In fact, dysregulated immune response, associated with inflammatory mediators, low-density lipoprotein (LDL), reactive oxygen species (ROS), increased calcium/phosphate levels and cyclic stretch can induce VICs to achieve an osteogenic and a pro-calcific profile, enhancing CAVD development [4, 5].

In other cardiovascular diseases, such as atherosclerosis, inflammation and adaptive immunity play a crucial role [6] and even aortic stenosis has been proposed as a chronic inflammatory process associated with atherosclerotic risk factors that accelerate the disease process, especially in patients with bicuspid aortic valve (BAV) [7, 8]. In fact, in mineralized bicuspid and tricuspid AV, it has been documented clonal expansions of T cell receptor repertoire, and presence of infiltrated T lymphocytes [9]. As a result, a growing focus on the function of infiltrating immune cells in the AV calcification has recently developed [10]. The presence of T cell infiltrate in CAVD, have initially been documented in calcification of porcine bioprostheses after AV replacement [11].

Later, transcriptomic analysis revealed an increased expression of various T lymphocyte-specific signalling and immune response-related pathways in calcified vs non-calcified AV [12]. According to current evidence, the pathogenic role of T cells in CAVD may depend on: i) their ability to increase inflammation and modulate the cytokine milieu, or ii) an antigen-specific T cell repertoire that drives a specific immunological response to CAVD-related antigens [13]. However, the role of T cells in CAVD pathophysiology still need to be clarified.

In this scenario, the involvement of the microbiota, especially gut microbiota (GM), should be considered. Host-microbiota interactions involving inflammatory and metabolic pathways seems to play a role in cardiovascular diseases [14]. Indeed, infections and endogenous microbiota products are able to promote atherosclerosis progression by eliciting local and systemic immune responses [15]. Moreover, increasing data suggest that microbiota-derived metabolites, such as trimethylamine N-oxide (TMAO), can contribute to atherosclerotic events [16] and high TMAO levels have been related with cardiac fibrosis exacerbation and left ventricular remodeling [17]. In addition, TMAO is able to induce adverse reactions such as the lipids oxidation, dysregulated activation of immune cells and inflammation worsening [18, 19].

Different studies have related circulating levels of phenylacetylglutamine, especially produced by Bifidobactariaceae members, with risk of coronary artery disease [20].

Moreover, the indoxyl sulfate, a GM-derived metabolite of amino acids, resulted to be a marker of atherosclerosis and aortic calcification [21]. Therefore, an increase in GM-derived metabolites can induce vascular smooth muscle cells or endothelial cells and trigger vascular calcification. Finally, in vivo, a reduction of short-chain fatty acids (SCFAs), the fermentation products of intestinal microorganisms, inhibits the G protein-coupled receptor pathway and increases the expression of inflammatory factors, resulting in lipid metabolism disorder, vascular remodelling, acceleration of arterial thrombosis, the onset and worsening of atherosclerosis, hypertension, and pulmonary fibrosis [22–24]. Notably, the alterations of gut microbial community have been documented in cardiovascular diseases and specific bacterial taxa have been identified as potential therapeutic target for cardiac valve calcification and coronary artery disease [25].

Still many questions remain to be answered in order to understand how the microbiota should contribute to CAVD development and, even if different studies documented a local shared role of bacteria-immunity interplay in atherogenesis, the potential impact of the microbiota-immunity axis in local CAVD inflammation is still unexplored.

In this study, we aimed to investigate and characterize the presence of valvular microbiota and the associated immune response in human CAV samples originating from two European populations.

## RESULTS

### Patients

We enrolled twenty patients (9 Italians and 11 Germans) with a confirmed risk factor for CAVD development showing a median age of 74 years (range 61–84) and a male to female ratio of 16:4, according to the higher prevalence in the male sex [26]. In addition, they had a high cardiovascular risk, defined by the presence of at least two risk factors among atrial fibrillation (AF), dyslipidaemia, diabetes, or smoking. A coronary artery disease (CAD) was detected in 13 out of 20 CAVD patients.

According to the New York Heart Association (NYHA) functional classification for heart failure 60% were assigned to NYHA class II, 30% to NYHA class III and the remaining 10% to NYHA class I [27].

Additionally, 45% of patients showed a BAV, which may be correlated with an early onset of CAVD symptoms (already around 60 years old).

Of note, the degree of total calcification of the CAV samples was 75% severe and 25% mild; in detail, resulted severe in 9 out of 11 German patients and in 6 out of 9 of Italian CAV.

Demographical and clinical patients' characteristics are reported in **Table 1**.

**Table 1.** Clinical information of the enrolled Italian (I) and German (G) patients.

**Patient ID**	**Age**	**Gender**	**Hypercholest erolemia**	**Diabetes**	**CAD**	**NYHA class**	**Calcification degree**	**BAV**	**AF**	**Smoke**
I 1	84	M	Yes	No	Yes	II	Mild	No	Yes	Yes
I 2	61	F	No	No	No	III	Severe	Yes	Yes	No
I 3	79	M	Yes	No	Yes	II	Severe	Yes	Yes	Yes
I 4	74	M	Yes	Yes	Yes	II	Severe	Yes	No	No
I 5	80	M	Yes	No	Yes	III	Severe	No	Yes	Yes
I 6	79	F	Yes	No	Yes	II	Mild	No	No	No
I 7	65	M	No	No	No	III	Severe	Yes	No	No
I 8	83	M	Yes	No	Yes	II	Mild	No	No	No
I 9	79	F	Yes	No	No	II	Severe	Yes	No	No
G 10	66	M	Yes	No	No	III	Severe	No	Yes	No
G 11	64	M	Yes	No	Yes	II	Severe	Yes	No	No
G 12	62	M	No	No	Yes	II	Severe	No	Yes	Yes
G 13	79	M	Yes	No	Yes	II	Mild	No	No	No
G 14	74	M	Yes	No	Yes	II	Severe	Yes	Yes	Yes
G 15	73	M	No	No	No	II	Mild	No	No	No
G 16	71	F	Yes	Yes	No	II	Severe	Yes	Yes	Yes
G 17	76	M	Yes	No	Yes	III	Severe	No	No	No
G 18	66	M	No	Yes	No	I	Severe	Yes	Yes	Yes
G 19	71	M	Yes	No	Yes	III	Severe	Yes	Yes	Yes
G 20	78	M	Yes	No	Yes	I	Severe	No	Yes	No

CAD= coronary artery disease; NYHA= New York Heart Association; BAV= bicuspid aortic valve; AF=atrial fibrillation.

### T cell lymphocytes infiltrate calcific aortic valves

To evaluate the presence of a local immune response, we expanded and cloned in vivo-activated infiltrating T cells. We obtained T cell clones (Tcc) from 17 out of 20 samples (85%), in detail from 100% of German patients' CAV samples and 67% from the Italian patients.

We obtained a total 204 T cell clones and in detail, 155/204 (76 %) were CD4^+^(T helper), 19 % was CD8^+^ (T cytotoxic) and 10/204 were both CD4 and CD8 negative.

Among the two patients' cohorts, Italians and Germans, they showed a similar distribution in Th clones (respectively, 85% vs 70%; p=0.145) and in CD4^-^CD8^-^ T cells (5% vs 5%, p=0.703). Noteworthy, a difference was found in T cytotoxic clones (10% vs 25%, p=0.045) that resulted more frequent in German patients.

### Profile of CAV associated microbiota

To assess the bacterial CAV composition, we sequenced a total of 1,064,815.00 reads for 20 samples.

**Figure 1:**
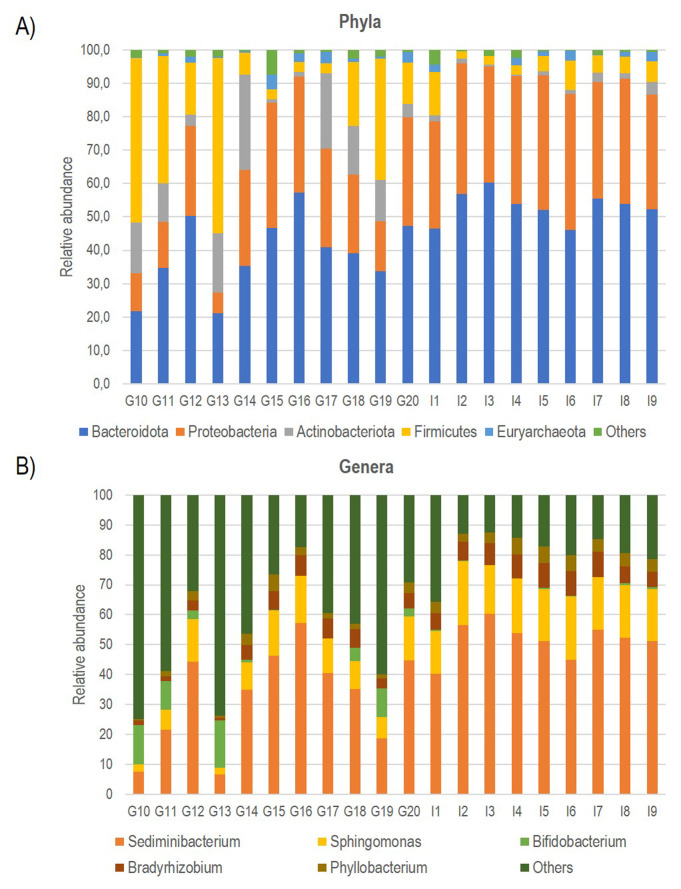
FIGURE 1: Bar plot showing the relative abundance of the five most represented Phyla (A) and Genera (B) documented in all CAV samples.

Consequently, using bioinformatics-based removal of human amplicons and following all pre-processing processes (pair merging, trimming, quality filtering, and chimera identification) a total of 483,253.00 (42%) were available for further analysis. The amplification of V3-V4 regions from the DNA in negative controls was unsuccessful, implying the overall sterility of the procedure. As expected, the first result obtained from the 16S rRNA gene sequencing analysis was a significant off-target amplification of human DNA. Indeed, an average of 46,8% of all ASV (amplicon Sequence Variants) detected in CAV samples were aligned to the human genome. This included the most prevalent ASV, which was further identified using BLAST as the *Homo sapiens haplogroup* with 100% identity with the human chromosomes. Consequently, we removed the human reads from the analysis and show that the specimens were still sufficiently sampled. The two rarefaction curves revealed the differences before and after the not aligned ASVs were removed (**Figure S1**). The subsequent taxonomic analysis, detailed in **Table 2**, reveals the presence of 17 phyla (>98% reads), 25 classes (>96% reads), 63 orders (>98% reads), 110 families (>99% reads) and 246 genera (>97% reads). Bacteroidota (45.3%) was the predominant phylum followed by Proteobacteria (29.6%), Firmicutes (14.4%) Actinobacteroidota (7.3%), and Euryarchaeota (1.6%). The most prevalent families were Chitinophagaceae, Sphingomonadaceae and Bifidobacteriaceae. Finally, the most abundant genera were *Sediminibacterium, Sphingomonas Bifidobacterium* and *Bradyrhizobium*; notably, the first accounting for a relative abundance of more than 40% (**Figure 1**).

In addition, in order to evaluate if specific clinical conditions such as BAV, CAD, AF, smoke and among demographical parameters could impact on CAV microbiota composition identify different patient profiles, we performed different PCoA analyses, but no significant differences emerged (**Figure S2**).

**Table 2.** Taxonomic analysis

**Rank**	**Reads %**	**Total ASV**	**Assigned ASV**
Phylum	0.983739	17	17
Class	0.961538	25	25
Order	0.984375	63	63
Family	0.990991	110	110
Genus	0.979757	246	242

**Figure 2:**
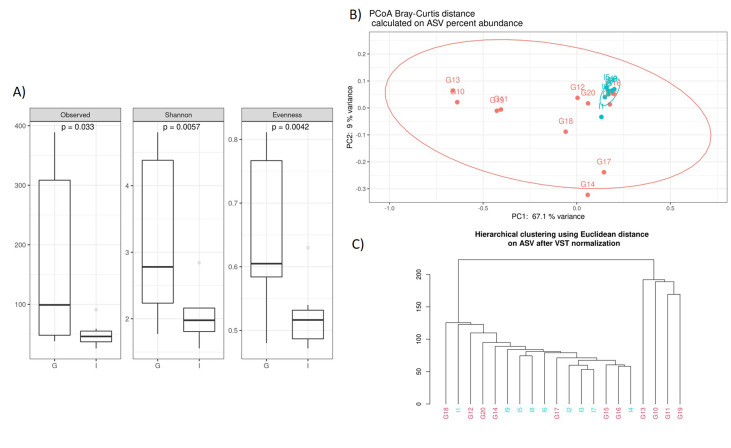
FIGURE 2: Box-plots (A) showing alpha diversity indices (Observed ASV richness, Shannon index, Pielou's Evenness) in German (G) and Italian (I) patients. Statistical differences were assessed using Mann-Whitney test and p-values less than 0.05 were considered statistically significant. Principal Coordinate Analysis (B) and Hierarchical clustering (C) of Italian (I) and German (G) CAV samples.

Noteworthy, we documented some interesting differences in CAV samples stratified by nationality. The alpha diversity analysis, performed using the observed species index (p=0,033), Shannon index (p=0,005), and Evenness (p=0,004), indicated that the German CAV samples had a higher number of microbial species and a greater diversity respect to the Italian patients' group (**Figure 2A**). Furthermore, Beta diversity was evaluated to estimate the difference between groups, and as showed by the PCoA analysis and the hierarchical clustering, the German and Italian samples formed separate clusters, with a significant separation (PERMANOVA p=0,005) (**Figure 2B, 2C)**) as suggested by the evaluation of the Bray-Curtis distance.

**Figure 3:**
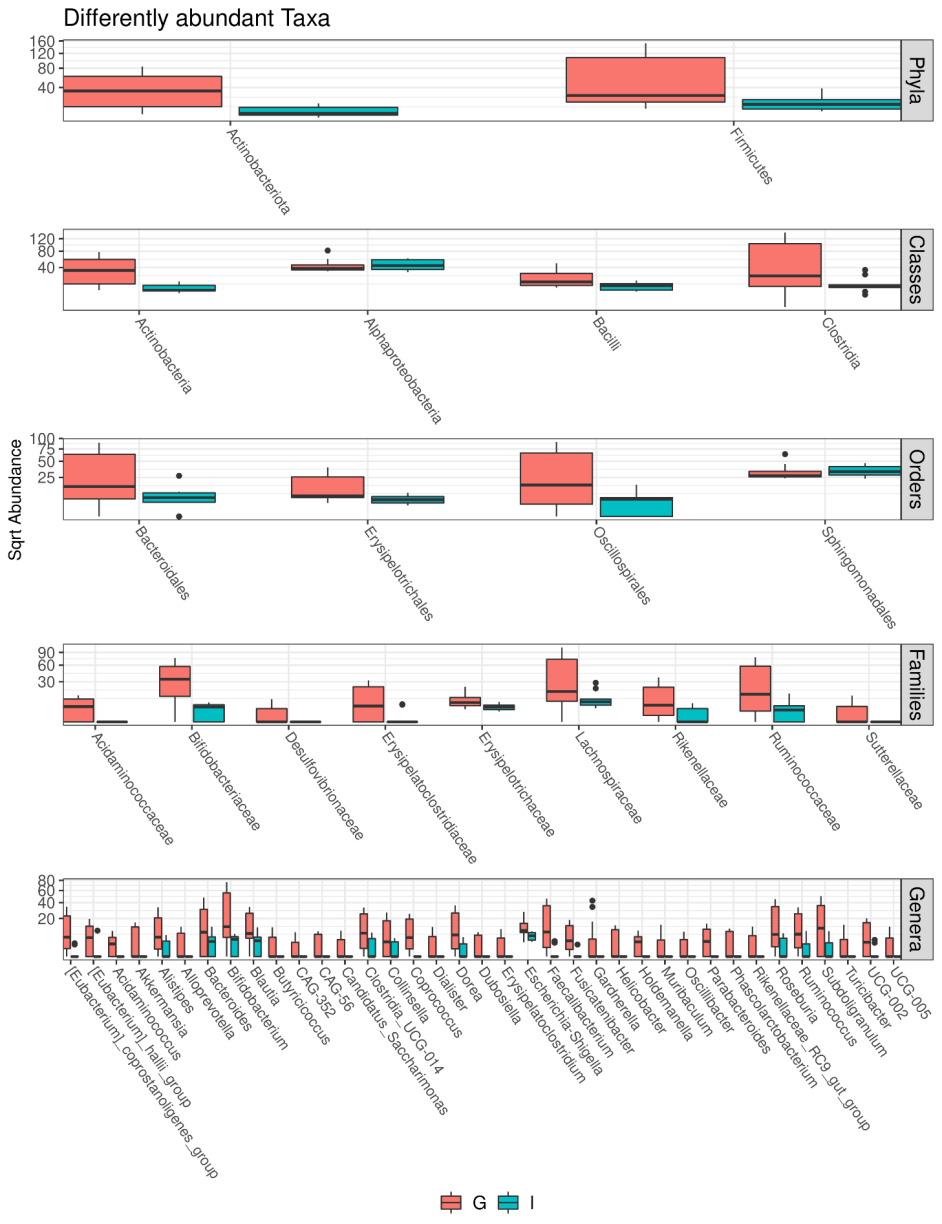
FIGURE 3: Box blot showing the significant differentially abundant taxa among German (G) and Italian (I) samples at phylum (A), Class (B), Order (C), Family (D) and Genus (E) level. Adjusted p values less than 0.05 were considered significant.

Multivariate analysis was performed to identify bacterial taxa that differed significantly between the two groups. Interestingly, German CAV samples showed higher abundance of several taxa compared to Italian CAV samples. The relative abundance of the top 5 bacteria at the phylum level isolated from Italian and German CAV samples is reported in **Figure 1** while the significant differentially distributed taxa are reported in **Figure 3** and **Table S1**.

### Correlation between infiltrating T cells and CAV microbiota

**Figure 4:**
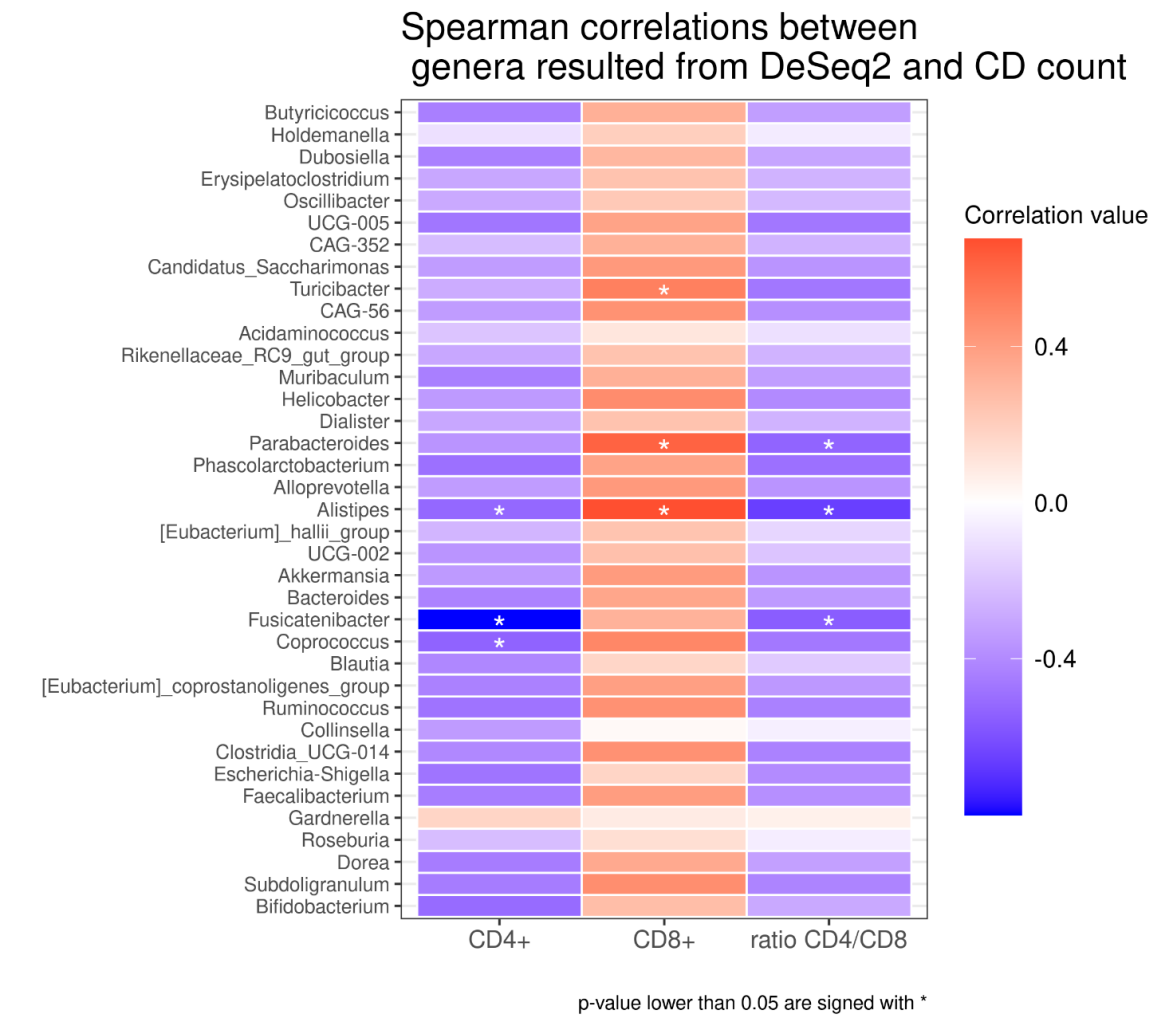
FIGURE 4: Heatmap of Spearman correlation between the differential abundant genera and Tcc expressions (CD4+, CD8+, ratio CD4/CD8). Colours indicate the rho of Spearman's coefficient and the asterisk (*) denotes a correlation with p value lower than 0.05. Red shades indicate positive correlations, whereas blue shades indicate negative correlations. (Tcc= T cell clones).

As reported in the **Figure 4**, we found different associations between the absolute number of T cell clones and bacterial taxa. Using Spearman's correlation coefficient, we found that several taxa negatively correlated with the total number of CD4^+^ Tcc, in particular with: the genus of *Coprococcus* (rho= -0,539; p= 0,038), *Fusicatenibacter* (rho=-0,799; p=0,000), *Alistipes* (rho=-0,522; p= 0,045), while the total number of CD8^+^ Tcc positively correlated with the genera of *Turicibacter* (rho=0,514; p= 0,049), *Parabacteroides* (rho=0,614; p=0,014) *Alistipes* (rho=0,673; p=0,005).

Finally, three genera exhibit an anti-correlation with the CD4/CD8 ratio, in particular *Parabacteroides* (rho= -0,535; p=0,039), *Alistipes* (rho= -0,661; p= 0,007), *Fusicatenibacter* (rho= -0,561; p= 0,029).

### Potential metabolic pathway CAV-microbiota correlated

The functional and metabolic changes in the bacterial CAV communities of patients were studied by inferring the metagenomes from the 16sRNA data and predicting the potential functions of the valve microbiota using the PICRUSt 2 software. Based on MetaCyc metabolic pathways' database (www.metacyc.org)*,* we found 161 predicted functional categories, with a significantly different abundance (LDA score >2.0) in the CAV microbiota among German and Italian patients (**Table S2**).

Interestingly, many predicted pathways were involved in amino acids metabolism, such like L-valine (p= 0,004), L-tryptophan (p=0,008), L-isoleucine (p=0,044), L-histidine (p= 0,002), L-methionine (p=0,002), L-lysine (p=0,016), L-glutamate and L-glutamine biosynthesis (p= 0,006). Moreover, different pathways, like PWY-7323 (p= 0,003) and PWY-5659 (p= 0,004) were related with the GDP-mannose biosynthesis.

## DISCUSSION

The calcific aortic valve disease is a pathophysiological process characterize by progressive stages involving the immune response, fibrotic tissue remodelling and calcification process [10, 28].

To date, the fine CAVD pathologic development is still poorly understood, but as for other CVD, the involvement of gut microbiota has been hypothesized [25, 29, 30].

In the present study, we explore for the first time, the potential role of CAV-associated microbiota and this interplay with infiltrating T cell in CAVD patients.

Firstly, in our cohort of patients of both nationalities (Italian and German), we confirmed the presence of infiltrating T cells in the majority of excised CAV, also documenting the prevalence of T helper subtype. In agreement with these results, the presence of a local adaptive immune response in CAV tissues is well established and correlated with faster disease progression [31, 32].

Hence, given the ability of T helper cells to either promote and coordinate both cellular and antibody specific immune responses (by helping macrophages and B lymphocytes respectively), the CD4^+^ overexpression in CAV samples can be explained by a local chronic inflammation condition, stimulating a unremitting immune activation [33, 34]. In addition, the number of infiltrating cytotoxic T cells in German patients was significantly higher compared to Italians. Remarkably, the *in vitro* role of CD8^+^ has been shown to stimulate osteoclast differentiation and the CD8^+^ cell-derived IFN-γ suppresses the calcium reabsorption potential of osteoclasts and thus could promote calcification in CAVD [35, 36]. Therefore, the elevated number of CD8^+^ founded in German patients could be predictive of a mayor grade of calcification.

Anyways, the most relevant result of our study is that for the first time we documented and characterized a CAV-associated microbiota.

Of note, as expected being very low the suspected microbial biomass, we worked to avoid environmental contamination as demonstrated by the unsuccessful amplification of negative controls samples. Moreover, the 16S rRNA sequencing showed a very high percentage (average of 50%) of ASVs aligned to the human genome. This off-target amplification has been previously described by Walker et al. and seems to be associated with the use of primer pair targeting the V3–V4 hypervariable regions [37]. In fact, if this commonly-used primer set for 16S rRNA sequencing identifies more taxa and displays higher diversity than the V1-V2 region [38], on the other hand, it results particularly susceptible to generate artefacts in samples containing an overwhelming ratio of human to bacterial DNA [39]. In fact, the off-target amplification has never been found in samples with lower levels of host DNA (e.g. fecal samples) [39–41], but CAV samples, where over the 97% of the extracted DNA is of host origin, can critically impact on this kind of analysis. Therefore, our data suggest that the use of primers targeting the V1–V2 region will be preferable for the amplification of human CAV samples [37, 42].

Anyway, after the removal of human reads, we found a consistent number of ASVs assigned to bacterial domains allowing the characterization of the valve associated microbiota in all CAV samples. The taxonomic phylum composition of CAV-associated microbiota, revealed that the most represented phylum was Bacteroidota, followed by Proteobacteria, Firmicutes, Actinobacteroidota, and Euryarchaeota. The large amount of Proteobacteria (about 30%) was similar to what previously observed in mitral valves [29] and atherosclerotic plaques[43]. The most prevalent families were Chitinophagaceae, Sphingomonadaceae and Bifidobacteriaceae. Finally, the most abundant genera were *Sediminibacterium, Sphingomonas Bifidobacterium* and *Bradyrhizobium*. Interestingly, *Sediminibacterium* (phylum Bacteroidota, family Chitinophagaceae) accounting for an average relative abundance of 40%, was previously documented, in addition to other environmental bacterial species with unknown pathogenicity, in atherosclerotic plaque and abdominal aortic aneurysmal wall biopsies from patients with chronic periodontitis [44–46]. Moreover, the decrease of this genus in the gut microbial composition of patients with CVDs has been documented [47].

Exploring if CAV microbiota composition could reflect patients' characteristics, we stratified the CAV sample according to clinical and demographical parameters, including BAV, CAD, AF, smoke habits and nationality. Surprisingly we documented a significant divergence only in CAV microbiota of patients from different countries. Interestingly, among the two groups, we found a different CAV-associated microbiota structure with German characterized by higher richness and biodiversity. So far, a lot of differences were observed in the taxonomic composition. Indeed, at phylum level, the samples from German patients showed higher levels of Actinobacteria and Firmicutes. Interestingly, elevated levels of circulating Actinobacteria were previously found in patients with CVD [48].

Moreover, at family taxonomic rank, German CAV reported significant higher abundances in Bifidobacteriaceae, Acidaminococcaceae, Lachnospiraceae, Ruminococcaceae, Rikenellaceae and Desulfovibrionaceae than Italian samples.

Finally, compared to Italian samples, German CAVs also displayed significant higher levels of several genera, including *Parabacteroides, Alloprevotella, Helicobacter, Alistipes and Ruminococcus*, described in literature as potential pathogens for CVD. Among this, the genus Helicobacter, and especially the *Helicobacter pylori*, strain has been previously described as a determinant for the development of cardiovascular events through the activation of inflammatory mediators, release of toxins, abnormal lipid metabolism and induction of autoimmune reactions [49]. Of note, also the genus *Alistipes*, was associated with some cardiovascular diseases such as AF, congestive heart failure, and atherosclerosis [50].

While *Clostridium spp.* and *Holdemanella spp*. were enriched in the intestinal microbiota of CAD patients [51–56].

In addition, the genus *Clostridium* has been already documented in valve disease, especially in endocarditis [57]. Several clostridial endocarditis have been reported and the majority being caused by *C. perfringens* excepted for a study reporting a rare prosthetic valve endocarditis due to *Clostridium bifermentans* infection [58].

In conclusion, our data suggested that the CAV-associated microbiota of German patients showed an enrichment of species promoting the CVD. Could be interesting to explore if these data have epidemiological links with country lifestyle.

Finally, we also evaluated the microbiota function using the PICRUSt2 software, a metagenome prediction tool comparing the significance of the differential abundance of predicted functional gene profiles to those from the bacterial 16S rRNA gene DNA sequencing [59]. The potential links between the CAV microbiota and metabolic profile may be useful to predict future CVD events.

Interestingly, it has been shown that the association of bacterial pathways connected to the amino acid metabolism, such as valine, L-tryptophan, isoleucine, histidine, methionine lysine, -glutamate and -glutamine biosynthesis is expressed both in Italian and German patients. This metabolic remodelling is an integral part of the pathogenesis of heart failure and has been correlated with the diagnostic profile of CVDs, such as the mitral valve regurgitation and stenosis [60, 61]. In addition, several studies shown that amino acids metabolic pathway is altered in patients with CAV stenosis [62, 63]. Notably, the Italian patients showed the expression of PWY-7323 related to GDP-mannose biosynthesis, a pathway already described in a work by Kurilshikov *et al.*, and negatively correlated with the CVD metabolic risk score [64].

Hence, all these results suggest potential applications of different microbiome-targeting approaches to modulate some taxa or bacterial pathway, for example, through personalized dietary control. Probiotics and prebiotics, thanks to the ability of modulating SCFA levels, may be a viable therapeutic option for cardiovascular disorders [65–68]. For instance, prebiotic supplementation was shown to improve endothelium-dependent vasodilation, systemic inflammation, and plasma propionate levels in stable coronary artery disease patients [69].

Although the PICRUSt analysis showed some features increased in German and others in Italian patients' group, detailed information about the pathway is limited, due to the limitations of 16sRNA, which restricts data interpretation in terms of genera level; so, metatranscriptomics sequencing technology is necessary for a deeper investigation.

Anyways, a potential explanation for the observed groups' differences can rely on the diverse Italian vs German lifestyle. In particular, the diet, represents one of the most important microbiota modifying element and German habits include a typical “Western diet” notoriously characterized by a high intake of saturated fats, omega-6 fatty acids, salt and refined sugars which can damage the heart and the immune system [70]. Moreover, German men usually eat two times more meat than women, increasing TMAO metabolites levels (associated with an increased heart disease), supporting the notable correlation between male sex and CAVD [71]. Of note, in 2019, Germany recorded the highest number of CAVD deaths in Europe [72].

On the other hand, the Italian adherence to the Mediterranean diet, represents a well-known protection against the CVD development, due to its anti-inflammatory properties [73–75].

So far, we seek for a potential association between CAV-associated microbiota and the adaptive immune response. Interestingly, some bacterial taxa showed a correlation with infiltrating T cells, suggesting how their abundance could modulate the immune balance. Specifically, we documented a positive correlation among CD8^+^ with the genera *Parabacteroides* already identified in rats with hypertensive heart failure, and with *Turicibacter,* described increased in gut microbiota of patients with hypertension [76, 77].

Interestingly, the genus *Alistipes* positively correlated with CD8^+^, but negatively with the CD4/CD8 ratio and with CD4^+^. This genus has been linked with CVD risk factors such as atherosclerosis, AF and hypertension [54] and was more abundant in samples from German patients, were the CD4/CD8 ratio is lower compared to Italian. Interestingly, a longitudinal study conducted on seropositive patients, showed how the inversion of CD4/CD8 ratio (<1) is associated with carotid Intima-media thickness progression, a documented CAD marker [78].

In conclusion, despite the present study having some limitations such as the restricted number of enrolled patients, the absence of non-calcific AV samples as a control group and the low taxonomical resolution of V3-V4 16S rRNA regions, we documented for the first time, the presence of a CAV-associated microbiota. Hence, even if we are currently unable to establish if bacterial DNA found in the CAV samples are molecules entangle in the calcific sample or if it denotes the presence of active resident microorganisms that have reached the aortic leaflets by the bloodstream, our findings could represent the fil rouge -currently missing- to explain the link with the immunity and the AV calcification process.

Nowadays, the mainstay for CAVD treatment is based on heart surgery with implantation of a valve prosthesis or by the transcatheter aortic valve implantation (TAVI), therefore this knowledge, if confirmed in studies including more patients, might pave the way for novel diagnostic and therapeutic options possibly based on microbiota shaping and so on personalized medicine approaches.

## MATERIALS AND METHODS

### Study Population and sample collection

Patients with CAVD undergoing scheduled surgical replacement of the calcified AV (CAV) were enrolled at Careggi Hospital (Florence, Italy) and Charitè Campus Virchow-Klinikum (Berlin, Germany). We excluded patients with inflammatory bowel or autoimmune diseases, and valve endocarditis acute infection. All enrolled patients provided written informed consent. Immediately after excision, CAV tissues were dissected into two parts: one was freshly used for immunologic analysis, the other one frozen (-80°C) until DNA extraction.

### Ethics statement

The study was conducted according with the Declaration of Helsinki and approved by the local ethics' committee “Comitato Etico Regionale per la Sperimentazione Clinica della Regione Toscana, Sezione AREA VASTA CENTRO” (15402_oss) and Campus Benjamin Franklin of Charitè (EA4/130/19).

### Analysis of tissue infiltrating lymphocytes

CAV samples were processed in order to isolate the tissue infiltrating T cells (TILs), as previously described [79]. Briefly, CAV were cultured for 7 days in RPMI 1640 medium supplemented with IL-2 (50 U/ml) to expand in vivo-activated TILs. Specimens were then disrupted, and single T cell blast was cloned under limiting dilution, as previously we described [80]. Tcc surface markers' expression (CD3, CD4, CD8) was analyzed through the Attune NxT flow cytometer (Life Technologies).

### AVs Microbiota Characterization

Genomic DNA was extracted from CAV samples using the DNeasy PowerSoil Pro Kit (Qiagen, Hilden, Germany) [81]. All the steps were performed under sterility conditions in a laminar flow cabinet. To monitor contamination during nucleic acid extraction, two samples made by sterile DNase/RNase free water has been extracted with the same method, conditions and kit, as negative controls. Briefly, samples were homogenized with Tissue Lyser LT (Qiagen, Hilden, Germany) for 5 minutes at 30 Hz and total DNA was captured on a silica membrane in a spin column format, washed and eluted. The quality and quantity of extracted DNA was assessed using the NanoDrop ND-1000 (Thermo Fisher Scientific, Waltham, MA, USA) and the Qubit Fluorometer (Thermo Fisher Scientific, Waltham, MA, USA), respectively.

Extracted DNA samples were sent to IGA Technology Services (Udine, Italy) where amplicons of the variable V3–V4 region of the bacterial 16S rRNA gene were sequenced using a paired-end approach (2 × 300 cycles) on the Illumina MiSeq platform, according to the Illumina 16S Metagenomic Sequencing Library Preparation protocol.

Raw sequences were processed using QIIME2 2021.4 [82]. The sequencing primers were removed using Cutadapt tool; DADA2 tool was used to perform paired-end reads merging, filtering and chimeras removal steps after trimming nucleotides from forward and reverse reads based on the quality profiles (--p-trunc-len-f 241 and --p-trunc-len-r 201) [83].

Hence, ASVs (amplicon sequence variants) were generated, and the V-search tool was used for taxonomic assignment using the SILVA database (release 138) as reference, with a 0.99 identity threshold [84].

The PICRUSt 2 (phylogenetic investigation of communities by reconstruction of unobserved state) bioinformatic tool was used to predict the metagenomics content from the 16S rRNA sequencing data [85].

### Statistical Analysis

Statistical analyses on the bacterial communities were performed in R 4.1 (R Core Team, 2014) with the help of the packages phyloseq 1.36.0, DESeq2 1.32.0 and other packages satisfying their dependencies, in particular, vegan 2.5-7. Packages ggplot2 3.3.5, dendextend 1.15.1 and ggpubr 0.4.0 were used to plot data and results.

Shannon, Observed ASV richness, and Evenness indices were used to estimate bacterial diversity in each sample using the function estimate richness from phyloseq. The evenness index was calculated using the formula E = S/log(R), where S is the Shannon diversity index and R is the number of ASVs in the sample. Differences in all indices were tested using the Mann-Whitney test.

Hierarchical clustering analysis of entire communities were performed on proportional count data of ASVs after VST normalization. PCoA was performed on proportional count data of each sample, adjusted with square root transformation.

At the different taxonomic ranks, the differential analysis of abundance was performed with DESeq2 on raw ASVs data. Moreover, the software GraphPad Prism (v.5) was used for the statistical analysis of immunological data; in particular, differences between samples of Italian and German patients were assessed using Mann-Whitney test and p-values less than 0.05 were considered statically significant.

Spearman correlation coefficients were calculated to evaluate the association between variables; p-values were corrected for multiple comparisons using the Benjamini-Hochberg FDR procedure for all analyses except for exploratory analyses performed between bacterial taxa and immune parameters [86]. Differential abundances of predicted pathways by the group were determined and displayed using linear discriminant analysis (LDA) effect size (LefSe).

### Data Availability Section

The datasets presented in this study can be found in online repositories. The 16S rRNA sequence data have been deposited in the NCBI Sequence Read Archive (SRA) database (https://www.ncbi.nlm.nih.gov/geo/query/acc.cgi?acc=GSE202396) under the BioProject accession number kdobcwmizvsrvmb.

## SUPPLEMENTAL MATERIAL

Click here for supplemental data file.

All supplemental data for this article are available online at www.microbialcell.com.

## Abbreviations:

CAVD: – Calcific aortic valve disease,
AV: – aortic valve,
VICs: – valve interstitial cells,
LDL: – low-density lipoprotein,
ROS: – reactive oxygen species,
BAV: – bicuspid aortic valve,
GM: – gut microbiota,
TMAO: – trimethylamine N-oxide,
SCFAs: – short-chain fatty acids,
AF: – atrial fibrillation,
CAD: – coronary artery disease,
NYHA: – New York Heart Association,
Tcc: – T cell clones,
TAVI: – transcatheter aortic valve implantation,
CAV: – calcified AV,
TILs: – infiltrating T cells,
LDA: – linear discriminant analysis,
SRA: – Sequence Read Archive.
