# Supplementary material for: The first taxonomic and functional characterization of human CAVD-associated microbiota
Source: Microb Cell. 2023 Jan 13;10(2):36–48. doi: 10.15698/mic2023.02.791 (PMC9896411; doi:10.15698/mic2023.02.791)
Supplement: Supplementary file 1 [file mic-10-036-s01.pdf]

Supplemental Informaton

The first taxonomic and functional characterization of human 1 CAVD-associated microbiota

Lavinia Curini[1,#], Brunilda Alushi[2,#], Mary Roxana Christopher[3], Simone Baldi[1], Leandro Di Gloria[4], Pierluigi Stefano[5], Anna Laganà[5], Luisa Iannone[5],Herko Grubitzsch[6], Ulf Landmesser[7], Matteo Ramazzotti[4], Elena Niccolai[1,§], Alexander Lauten[2,§], Amedeo Amedei[1,8,§,*]

[1] Department of Clinical and Experimental Medicine, University of Florence, 50139 Florence, Italy.
[2] Department of Cardiology, Campus Benjamin Franklin, Charité Universitätsmedizin Berlin, and German Centre for Cardiovascular Research (DZHK); Department of Interventional Cardiology, Klinik Vincentinum Augsburg, Germany.
[3] Department of Cardiology, Campus Benjamin Franklin, Charité Universitätsmedizin Berlin, and German Centre for Cardiovascular Research (DZHK).
[4] Department of Biomedical, Experimental and Clinical Sciences "Mario Serio", University of Florence, Florence, Italy.
[5] Cardiac Surgery, Careggi University Hospital, 50134 Florence, Italy.
[6] Berlin Institute of Health; Department of Cardiology, German Heart Centre Berlin (DHZB).
[7] Department of Cardiology, Campus Benjamin Franklin, Charité Universitätsmedizin Berlin and German Centre for Cardiovascular Research (DZHK); Berlin Institute of Health.
[8] SOD of Interdisciplinary Internal Medicine, Azienda Ospedaliera Universitaria Careggi (AOUC), 50139 Florence, Italy.
[#] These authors contributed equally
[§] These senior authors contributed equally

* Corresponding Author:
Amedeo Amedei, Department of Clinical and Experimental Medicine, University of Florence, 50139 Florence, Italy;
E-mail: amedeo.amedei@unifi.it

TABLE S1. Differentially more abundant taxa abundant in German and Italian patients. Statistical differences were evaluated using the Mann-Whitney test and adj. p values less than 0.05 were considered significant.

Log2FoldChange	padj			
2,4972	3,09x10^{-6}	Actinobacteriota	**Phylum**	**German patients**
1,3891	0,0052	Firmicutes		
3,2629	9,8994	Actinobacteria	**Class**	**German patients**
2,8281	0,0124	Clostridia		
0,9048	0,0124	Bacilli		
6,8150	0,0124	Verrucomicrobiae		
3,0158	0,0035	Methanomicrobia		
6,2647	0,0124	Campylobacteria		
-0,7453	0,0124	Alphaproteobacteria		**Italian patients**
6,5425	7,53x10^{-8}	Bifidobacteriales	**Order**	**German patients**
2,9663	0,0001	Enterobacterales		
3,3535	0,0001	Erysipelotrichales		
3,9398	8,39x10^{-5}	Lachnospirales		
7,7445	0,0007	Acidaminococcales		
5,1654	0,0021	Oscillospirales		
8,1290	0,0021	Verrucomicrobiales		
7,4612	0,0045	Campylobacterales		
4,0268	0,0073	Bacteroidales		
3,083	0,0150	Methanomicrobiales		
4,4655	0,0196	Clostridia_UCG-014		
3,9409	0,0240	Coriobacteriales		
6,6471	0,0407	Saccharimonadales		
6,5704	0,0143	Desulfovibrionales		
-0,3770	0,0167	Sphingomonadales		**Italian patients**
6,6652	8,75x10^{-08}	Bifidobacteriaceae	**Family**	**German patients**
7,3634	2,6x10^{-05}	[Eubacterium]_coprostanoligenes_group		
3,1610	0,0003	Enterobacteriaceae		
7,9945	0,0003	Acidaminococcaceae		
7,9164	0,0003	Tannerellaceae		
4,1306	0,0004	Lachnospiraceae		
7,6843	0,0022	Helicobacteraceae		
5,6592	0,0024	Ruminococcaceae		
4,9017	0,0056	Clostridia_UCG-014		
4,8158	0,0056	Rikenellaceae		
8,3903	0,0074	Akkermansiaceae		
7,0884	0,0148	Marinifilaceae		
2,0129	0,0227	Erysipelotrichaceae		
5,1478	0,0328	Coriobacteriaceae		
4,2927	0,0344	Bacteroidaceae		
6,9123	0,03571	Saccharimonadaceae		
6,8487	0,0393	Desulfovibrionaceae		
5,1170	0,0422	Erysipelatoclostridiaceae		
4,2172	0,0451	Muribaculaceae		
6,6782	0,0451	Sutterellaceae		
6,2621	0,0451	Butyricicoccaceae		

25,3329	$2{,}19x10^{-15}$	*Gardnerella*	***Genus***	**German patients**
9,8417	$3{,}83x\ 10^{-07}$	*Coprococcus*		
6,3713	0,0009	*Bifidobacterium*		
7,4383	0,0018	*Eubacterium coprostanoligenes_group*		
7,4914	0,0018	*Fusicatenibacter*		
7,4438	0,0020	*Faecalibacterium*		
7,9725	0,0044	*Parabacteroides*		
5,9760	0,0050	*Lachnospiraceae*		
8,4173	0,0056	*Akkermansia*		
6,0677	0,0097	*Dorea*		
7,8339	0,0097	*Alloprevotella*		
7,7305	0,0097	*Helicobacter*		
6,3390	0,0105	*Subdoligranulum*		
7,0199	0,0105	*uncultured*		
7,4519	0,0117	*Phascolarctobacterium*		
7,1233	0,0173	*CAG-56*		
6,4660	0,0189	*Acidaminococcus*		
6,3311	0,0198	*Holdemanella*		
6,4762	0,0198	[*Eubacterium*] *hallii group*		
5,0820	0,0225	*Roseburia*		
6,7808	0,0225	*UCG-005*		
2,3252	0,0242	*Escherichia-Shigella*		
6,6054	0,0242	*Dubosiella*		
5,8625	0,0250	*UCG-002*		
4,9910	0,0278	*Clostridia UCG-014*		
4,9683	0,0279	*Ruminococcus*		
6,9334	0,0279	*Rikenellaceae_RC9_gut_group*		
6,3758	0,0280	*Muribaculum*		
6,9344	0,0286	*Candidatus_Saccharimonas*		
6,2962	0,0287	*Turicibacter*		
6,2352	0,0287	*Butyricicoccus*		
6,7315	0,0301	*Dialister*		
5,2167	0,0318	*Collinsella*		
4,6504	0,0318	*Alistipes*		
6,4889	0,0385	*Oscillibacter*		
6,4515	0,0407	*CAG-352*		
3,7252	0,0431	*Blautia*		
4,3385	0,0450	*Bacteroides*		
6,1252	0,0470	*Erysipelatoclostridium*		

TABLE S2. Potential function of the valve microbiota in German and Italian patients with LDA score >2.0. LDA= Linear discriminant analysis.

Pathway	Log_highest_mean	Class_with_highest_mean	logLDA_score	p-value	MetaCyc_ID
methylgallate_degradation	3,03	Italy	2,30	0,001	METHYLGALLATE-DEGRADATION-PWY
glycine_betaine_degradation_I	3,16	Italy	2,45	0,009	PWY-3661
CMP_legionaminate_biosynthesis_I	2,63	Germany	2,37	0,047	PWY-6749
TCA_cycle_IV_2_oxoglutarate_decarboxylase_	3,80	Italy	2,85	0,003	P105-PWY
superpathway_of_glycolysis_and_Entner_Doudoroff	3,54	Italy	2,48	0,004	GLYCOLYSIS-E-D
superpathway_of_glucose_and_xylose_degradation	3,60	Germany	2,67	0,003	PWY-6901
tetrapyrrole_biosynthesis_I_from_glutamate_	3,75	Italy	2,70	0,002	PWY-5188
homolactic_fermentation	3,67	Germany	3,04	0,001	ANAEROFRUCAT-PWY
TCA_cycle_VIII_helicobacter_	3,94	Italy	2,91	0,003	REDCITCYC
colanic_acid_building_blocks_biosynthesis	3,69	Italy	2,54	0,006	COLANSYN-PWY
L_leucine_degradation_I	3,65	Italy	2,87	0,003	LEU-DEG2-PWY
superpathway_of_glyoxylate_bypass_and_TCA	3,60	Italy	2,77	0,003	TCA-GLYOX-BYPASS
superpathway_of_heme_biosynthesis_from_glycine	3,54	Italy	2,77	0,001	PWY-5920
L_isoleucine_biosynthesis_I_from_threonine_	4,09	Italy	2,87	0,004	ILEUSYN-PWY
superpathay_of_heme_biosynthesis_from_glutamate	3,68	Italy	2,75	0,004	PWY-5918
superpathway_of_menaquinol_7_biosynthesis	2,81	Germany	2,17	0,037	PWY-5840
adenine_and_adenosine_salvage_III	3,73	Germany	2,52	0,025	PWY-6609
superpathway_of_L_aspartate_and_L_asparagine_biosynthesis	3,40	Germany	2,85	0,030	ASPASN-PWY
ubiquinol_8_biosynthesis_prokaryotic_	3,80	Italy	3,01	0,002	PWY-6708
1_4_dihydroxy_6_naphthoate_biosynthesis_I	3,47	Italy	2,66	0,011	PWY-7374
superpathway_of_hexuronide_and_hexuronate_degradation	3,55	Italy	2,53	0,000	GALACT-GLUCUROCAT-PWY
superpathway_of_beta_D_glucuronide_and_D_glucuronate_degradation	3,52	Italy	2,47	0,001	GLUCUROCAT-PWY
NAD_salvage_pathway_II	2,71	Germany	2,16	0,002	NAD-BIOSYNTHESIS-II

cis_vaccenate_biosynthesis	3,95	Italy	2,62	0,002	PWY-5973
superpathway_of_fucose_and_rhamnose_degradation	2,71	Germany	2,33	0,014	FUC-RHAMCAT-PWY
L_valine_biosynthesis	4,09	Italy	2,87	0,004	VALSYN-PWY
superpathway_of_demethylmenaquinol_8_biosynthesis	2,68	Germany	2,07	0,044	PWY-5861
superpathway_of_UDP_glucose_derived_O_antigen_building_blocks_biosynthesis	3,26	Germany	2,55	0,014	PWY-7328
superpathway_of_methylglyoxal_degradation	1,78	Germany	2,13	0,003	METHGLYUT-PWY
UDP_N_acetyl_D_glucosamine_biosynthesis_I	3,61	Germany	2,62	0,007	UDPNAGSYN-PWY
L_glutamate_and_L_glutamine_biosynthesis	3,50	Germany	2,93	0,007	PWY-5505
pyrimidine_deoxyribonucleotides_de_novo_biosynthesis_III	3,13	Germany	2,65	0,025	PWY-6545
gondoate_biosynthesis_anaerobic_	3,97	Italy	2,66	0,002	PWY-7663
methylphosphonate_degradation_I	3,34	Italy	2,52	0,044	PWY0-1533
glycolysis_I_from_glucose_6_phosphate_	3,77	Germany	2,66	0,007	GLYCOLYSIS
glyoxylate_cycle	3,49	Italy	2,68	0,003	GLYOXYLATE-BYPASS
superpathway_of_pyrimidine_ribonucleosides_salvage	3,47	Germany	2,86	0,030	PWY-7196
gallate_degradation_II	3,01	Italy	2,34	0,000	GALLATE-DEGRADATION-I-PWY
superpathway_of_histidine_purine_and_pyrimidine_biosynthesis	3,63	Germany	2,69	0,007	PRPP-PWY
Kdo_transfer_to_lipid_IVA_III_Chlamydia_	3,60	Italy	2,70	0,009	PWY-6467
4_deoxy_L_threo_hex_4_enopyranuronate_degradation	3,64	Italy	2,64	0,003	PWY-6507
ubiquinol_7_biosynthesis_prokaryotic_	3,80	Italy	3,01	0,002	PWY-5855
chorismate_biosynthesis_I	3,77	Germany	2,65	0,017	ARO-PWY
L_isoleucine_biosynthesis_IV	3,69	Germany	3,15	0,044	PWY-5104
sucrose_degradation_IV_sucrose_phosphorylase_	3,16	Germany	2,76	0,017	PWY-5384
flavin_biosynthesis_I_bacteria_and_plants_	3,74	Italy	2,31	0,002	RIBOSYN2-PWY
superpathway_of_purine_deoxyribonucleosides_degradation	3,42	Germany	2,63	0,037	PWY0-1297
fucose_degradation	2,67	Germany	2,30	0,011	FUCCAT-PWY
L_methionine_biosynthesis_III	3,83	Italy	2,74	0,002	HSERMETANA-PWY
superpathway_of_Kdo_2_lipid_A_biosynthesis	2,47	Germany	2,02	0,014	KDO-NAGLIPASYN-PWY
superpathway_of_pyrimidine_deoxyribonucleoside_salvage	3,63	Italy	2,34	0,006	PWY-7200

pyrimidine_deoxyribonucleotides_biosynthesis_from_CTP	2,90	Germany	2,45	0,001	PWY-7210
lipid_IVA_biosynthesis	3,64	Italy	2,74	0,009	NAGLIPASYN-PWY
O_antigen_building_blocks_biosynthesis_E_coli_	3,66	Germany	2,50	0,004	OANTIGEN-PWY
queuosine_biosynthesis	3,74	Italy	2,79	0,002	PWY-6700
hexitol_fermentation_to_lactate_formate_ethanol_and_acetate	2,60	Germany	2,23	0,002	P461-PWY
superpathway_of_L_tryptophan_biosynthesis	2,43	Germany	2,01	0,009	PWY-6629
superpathway_of_aromatic_amino_acid_biosynthesis	3,79	Germany	2,63	0,025	COMPLETE-ARO-PWY
L_isoleucine_biosynthesis_II	4,08	Italy	2,75	0,003	PWY-5101
GDP_D_glycero_alpha_D_manno_heptose_biosynthesis	3,53	Italy	2,72	0,007	PWY-6478
ubiquinol_9_biosynthesis_prokaryotic_	3,80	Italy	3,01	0,002	PWY-5856
L_ornithine_biosynthesis	3,74	Italy	2,28	0,037	GLUTORN-PWY
starch_degradation_V	3,84	Germany	2,86	0,011	PWY-6737
superpathway_of_menaquinol_6_biosynthesis_I	2,61	Germany	2,03	0,011	PWY-5850
dTDP_L_rhamnose_biosynthesis_I	3,90	Italy	2,39	0,020	DTDPRHAMSYN-PWY
peptidoglycan_maturation_meso_diaminopimelate_containing_	3,78	Italy	2,49	0,009	PWY0-1586
preQ0_biosynthesis	3,52	Italy	2,37	0,006	PWY-6703
superpathway_of_purine_nucleotides_de_novo_biosynthesis_II	3,69	Germany	2,85	0,003	DENOVOPURINE2-PWY
GDP_mannose_biosynthesis	3,79	Italy	2,62	0,004	PWY-5659
superpathway_of_L_alanine_biosynthesis	3,35	Germany	2,89	0,004	PWY0-1061
acetylene_degradation	3,44	Germany	3,02	0,003	P161-PWY
superpathway_of_pyridoxal_5_phosphate_biosynthesis_and_salvage	3,24	Germany	2,57	0,017	PWY0-845
oleate_biosynthesis_IV_anaerobic_	3,47	Italy	2,42	0,037	PWY-7664
superpathway_of_guanosine_nucleotides_de_novo_biosynthesis_I	3,79	Italy	2,66	0,037	PWY-7228
sulfate_reduction_I_assimilatory_	3,82	Italy	2,98	0,003	SO4ASSIM-PWY
vanillin_and_vanillate_degradation_I	3,03	Italy	2,33	0,000	PWY-7097
fatty_acid_salvage	3,86	Italy	3,06	0,007	PWY-7094
UMP_biosynthesis	3,85	Germany	2,55	0,025	PWY-5686
superpathway_of_thiamin_diphosphate_biosynthesis_II	3,11	Germany	2,67	0,009	PWY-6895
methanogenesis_from_acetate	2,83	Germany	2,39	0,006	METH-ACETATE-PWY
superpathway_of_N_acetylglucosamine_N_acetylmannosamine_and_N_acetylneuraminate_degradation	3,04	Germany	2,67	0,025	GLCMANNANAUT-PWY

tRNA_charging	3,77	Germany	2,39	0,044	TRNA-CHARGING-PWY
superpathway_of_GDP_mannose_derived_O_antigen_building_blocks_biosynthesis	3,72	Italy	2,74	0,003	PWY-7323
ADP_L_glycero_beta_D_manno_heptose_biosynthesis	3,56	Italy	2,75	0,009	PWY0-1241
D_galacturonate_degradation_I	3,66	Italy	2,60	0,003	GALACTUROCAT-PWY
fatty_acid_elongation__saturated	3,96	Italy	2,96	0,003	FASYN-ELONG-PWY
palmitate_biosynthesis_II_bacteria_and_plants_	3,28	Germany	2,63	0,020	PWY-5971
ubiquinol_10_biosynthesis_prokaryotic_	3,80	Italy	3,01	0,002	PWY-5857
superpathway_of_branched_amino_acid_biosynthesis	3,98	Italy	2,44	0,004	BRANCHED-CHAIN-AA-SYN-PWY
toluene_degradation_II_aerobic__via_4_methylcatechol_	2,99	Germany	2,20	0,017	PWY-5182
6_hydroxymethyl_dihydropterin_diphosphate_biosynthesis_I	3,76	Italy	2,45	0,002	PWY-6147
arginine_ornithine_and_proline_interconversion	3,17	Germany	2,44	0,020	ARGORNPROST-PWY
L_histidine_degradation_I	3,69	Italy	2,85	0,002	HISDEG-PWY
gallate_degradation_I	3,01	Italy	2,33	0,000	GALLATE-DEGRADATION-II-PWY
thiamin_salvage_II	3,61	Germany	2,34	0,014	PWY-6897
pyrimidine_deoxyribonucleotide_phosphorylation	3,71	Italy	2,73	0,003	PWY-7197
superpathway_of_glycol_metabolism_and_degradation	2,23	Germany	2,02	0,009	GLYCOL-GLYOXDEG-PWY
superpathway_of_ubiquinol_8_biosynthesis_prokaryotic_	3,77	Italy	2,96	0,003	UBISYN-PWY
superpathway_of_pyrimidine_deoxyribonucleosides_degradation	3,36	Germany	2,76	0,006	PWY0-1298
ppGpp_biosynthesis	3,34	Italy	2,47	0,037	PPGPPMET-PWY
aromatic_biogenic_amine_degradation_bacteria_	3,52	Italy	2,81	0,000	PWY-7431
protocatechuate_degradation_I_meta_cleavage_pathway_	3,05	Italy	2,34	0,000	P184-PWY
L_lysine_biosynthesis_II	2,84	Germany	2,32	0,017	PWY-2941
L_tryptophan_biosynthesis	3,77	Italy	2,32	0,003	TRPSYN-PWY
TCA_cycle_V_2_oxoglutarate_ferredoxin_oxidoreductase_	3,89	Italy	2,74	0,004	PWY-6969
phenylacetate_degradation_I_aerobic_	3,18	Italy	2,34	0,037	PWY0-321
guanosine_nucleotides_degradation_III	3,86	Italy	2,99	0,001	PWY-6608

guanosine_ribonucleotides_de_novo_biosynthesis	3,81	Germany	2,48	0,030	PWY-7221
polyisoprenoid_biosynthesis_E_coli_	3,74	Italy	2,45	0,037	POLYISOPRENSYN-PWY
L_glutamate_degradation_VIII_to_propanoate_	1,92	Germany	2,03	0,009	PWY-5088
superpathway_of_guanosine_nucleotides_de_novo_biosynthesis_II	3,78	Italy	2,61	0,009	PWY-6125
nylon_6_oligomer_degradation	2,79	Italy	2,10	0,011	P621-PWY
urate_biosynthesisorinosine_5_phosphate_degradation	3,86	Italy	2,71	0,001	PWY-5695
catechol_degradation_to_beta_ketoadipate	2,77	Italy	2,05	0,017	CATECHOL-ORTHO-CLEAVAGE-PWY
1_4_dihydroxy_6_naphthoate_biosynthesis_II	3,47	Italy	2,65	0,011	PWY-7371
superpathway_of_vanillin_and_vanillate_degradation	3,03	Italy	2,33	0,000	PWY-6338
formaldehyde_oxidation_I	3,17	Germany	2,69	0,001	RUMP-PWY
heterolactic_fermentation	3,31	Germany	2,54	0,017	P122-PWY
superpathway_of_sulfate_assimilation_and_cysteine_biosynthesis	3,80	Italy	2,73	0,002	SULFATE-CYS-PWY
glycolysis_III_from_glucose_	3,81	Germany	2,59	0,011	ANAGLYCOLYSIS-PWY
superpathway_of_menaquinol_10_biosynthesis	2,61	Germany	2,03	0,011	PWY-5896
reductive_acetyl_coenzyme_A_pathway	1,29	Germany	2,13	0,011	CODH-PWY
L_tyrosine_degradation_I	3,32	Italy	2,58	0,001	TYRFUMCAT-PWY
5Z_dodec_5_enoate_biosynthesis	3,47	Italy	2,57	0,006	PWY0-862
formaldehyde_assimilation_II_RuMP_Cycle_	3,34	Germany	2,87	0,001	PWY-1861
tetrapyrrole_biosynthesis_II_from_glycine_	3,76	Italy	2,80	0,002	PWY-5189
superpathway_of_menaquinol_8_biosynthesis_I	2,82	Germany	2,16	0,044	PWY-5838
aerobic_respiration_I_cytochrome_c_	4,21	Italy	3,25	0,025	PWY-3781
superpathway_of_thiamin_diphosphate_biosynthesis_I	3,42	Germany	2,76	0,037	THISYN-PWY
thiazole_biosynthesis_II_Bacillus_	2,67	Germany	2,32	0,009	PWY-6891
vanillin_and_vanillate_degradation_II	3,05	Italy	2,35	0,000	PWY-7098
D_fructuronate_degradation	3,69	Italy	2,45	0,004	PWY-7242
glycogen_degradation_I_bacterial_	3,76	Germany	2,88	0,009	GLYCOCAT-PWY
S_adenosyl_L_methionine_cycle_I	3,54	Germany	3,01	0,002	PWY-6151
TCA_cycle_I_prokaryotic_	3,90	Italy	2,81	0,004	TCA

heme_biosynthesis_I_aerobic_	3,66	Italy	2,82	0,003	HEME-BIOSYNTHESIS-II
adenosine_nucleotides_degradation_II	3,86	Italy	3,02	0,001	SALVADEHYPOX-PWY
pyrimidine_deoxyribonucleosides_salvage	3,66	Italy	2,24	0,011	PWY-7199
CMP_3_deoxy_D_manno_octulosonate_biosynthesis_I	3,69	Italy	2,82	0,003	PWY-1269
polymyxin_resistance	1,81	Germany	2,05	0,025	PWY0-1338
fatty_acid_beta_oxidation_I	3,85	Italy	3,04	0,007	FAO-PWY
pyruvate_fermentation_to_acetate_and_lactate_II	3,51	Germany	3,08	0,001	PWY-5100
toluene_degradation_I_aerobic__via_o_cresol_	2,99	Germany	2,20	0,017	PWY-5180
pyridoxal_5_phosphate_biosynthesis_I	3,14	Germany	2,50	0,025	PYRIDOXSYN-PWY
6_hydroxymethyl_dihydropterin_diphosphate_biosynthesis_III_Chlamydia_	3,77	Italy	2,52	0,009	PWY-7539
glycolysis_II_from_fructose_6_phosphate_	3,72	Germany	2,68	0,009	PWY-5484
protocatechuate_degradation_II_ortho_cleavage_pathway_	3,43	Italy	2,63	0,037	PROTOCATECHUATE-ORTHO-CLEAVAGE-PWY
1_5_anhydrofructose_degradation	2,67	Italy	2,03	0,011	PWY-6992
phosphopantothenate_biosynthesis_I	3,73	Italy	2,51	0,001	PANTO-PWY
thiazole_biosynthesis_I_E_coli_	3,32	Germany	2,94	0,044	PWY-6892
pyrimidine_deoxyribonucleotides_de_novo_biosynthesis_IV	2,78	Germany	2,36	0,001	PWY-7198
pyrimidine_deoxyribonucleotides_de_novo_biosynthesis_II	3,57	Germany	2,74	0,003	PWY-7187
pyruvate_fermentation_to_propanoate_I	3,84	Italy	2,88	0,003	P108-PWY
superpathway_of_N_acetylneuraminate_degradation	3,19	Germany	2,73	0,017	P441-PWY
chlorosalicylate_degradation	2,75	Italy	2,07	0,011	PWY-6107
heme_biosynthesis_II_anaerobic_	3,69	Italy	2,75	0,003	HEMESYN2-PWY
incomplete_reductive_TCA_cycle	3,59	Germany	2,99	0,006	P42-PWY
L_rhamnose_degradation_I	2,82	Germany	2,36	0,037	RHAMCAT-PWY
purine_nucleotides_degradation_II_aerobic_	3,65	Italy	2,51	0,002	PWY-6353
sucrose_degradation_III_sucrose_invertase_	3,43	Germany	3,05	0,017	PWY-621
glucose_and_glucose_1_phosphate_degradation	2,80	Germany	2,13	0,037	GLUCOSE1PMETAB-PWY

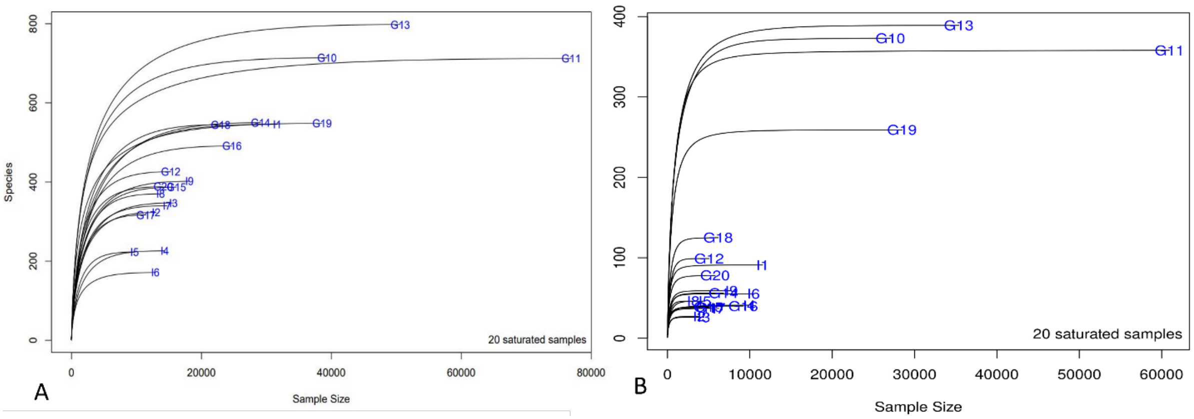

FIGURE S1: Rarefaction curves showing the level ASVs saturation. Panel A and panel B show respectively the rarefaction curves before and after the not aligned ASVs were removed. (ASV= Amplicon sequence variant)

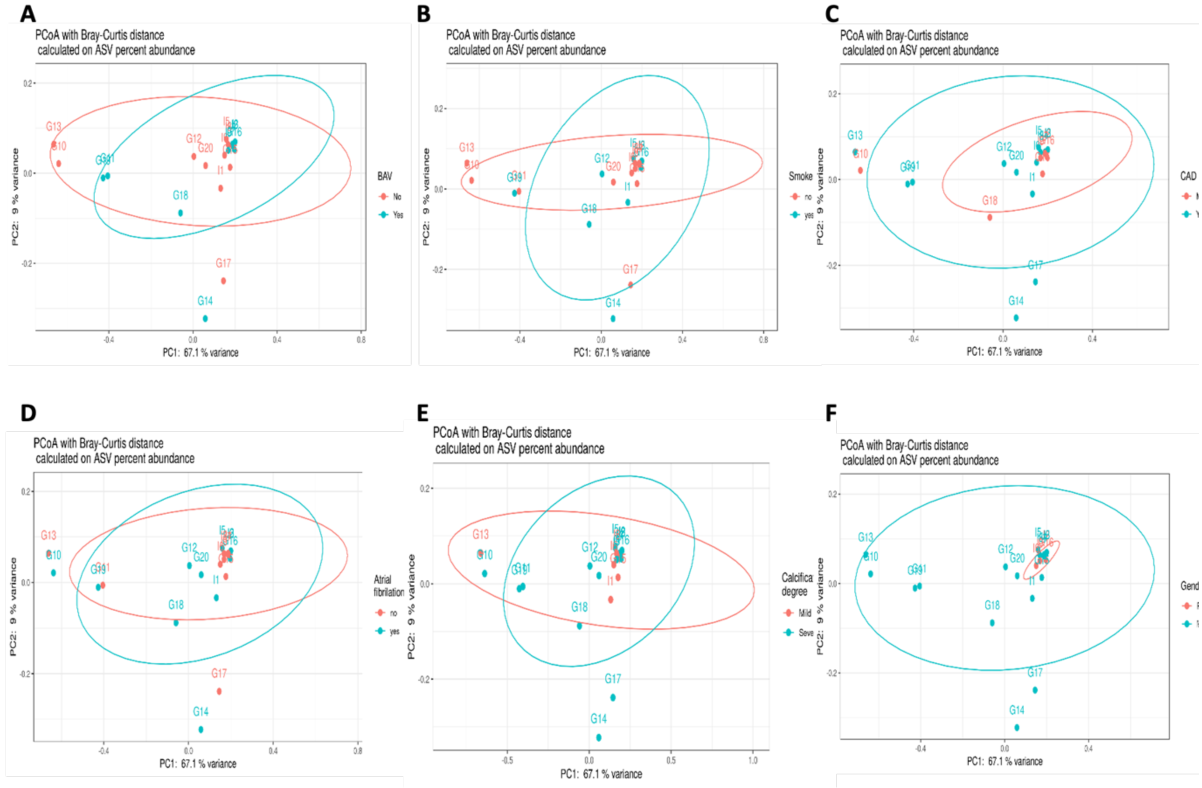

FIGURE S2: Principal Coordinate Analysis of Italian (I) and German (G) CAV samples. Panels from A to F represent the following clinical conditions: BAV, Smoke, CAD, AF, Calcification degree and Gender.
